# Primary intrathoracic gastric volvulus in the neonatal period: a differential diagnosis of esophageal atresia

**DOI:** 10.11604/pamj.2014.17.261.2650

**Published:** 2014-04-10

**Authors:** Driss El Azzouzi

**Affiliations:** 1 Department of Pediatric Surgery, Hospital of AL FARABI, University of Mohammed I, Oujda, Morocco

**Keywords:** Gastric volvulus, Newborn

## Abstract

Intrathoracic gastric volvulus in the neonatal period is a rare surgical emergency. Delays in diagnosis and treatment are life-threatening due to progressive deterioration of the gastric walls. Presentation in this period can be confused with the possibility of esophageal atresia or esophageal web. The upper gastrointestinal tract contrast study is diagnostic in this disease. The authors report a case of acute intrathoracic gastric volvulus diagnosis by radiologic-contrast-study in 1-day-old girl that was confirmed at surgery. The physiopathology, classification and different presentations of this entity are briefly reviewed.

## Introduction

Primary gastric volvulus in the neonatal period is extremely rare, potentially life-threatening condition, which is difficult to diagnose. This case of acute gastric volvulus in neonates with the aim of describing this rare disease and its varied clinical manifestations

## Patient and observation

**Figure 1:**
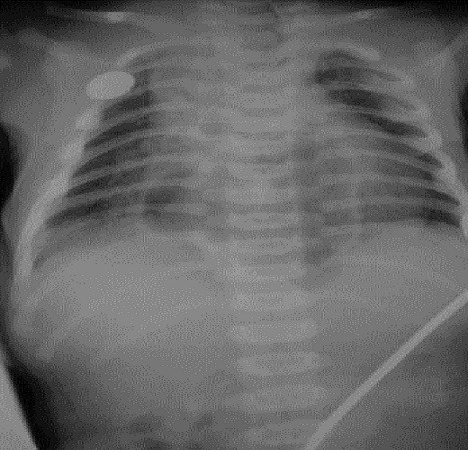
Neonatal chest X-ray showing catheter in esophagus at T6 vertebral level and intrathoracic gastric bubble in the mediastinum

**Figure 2:**
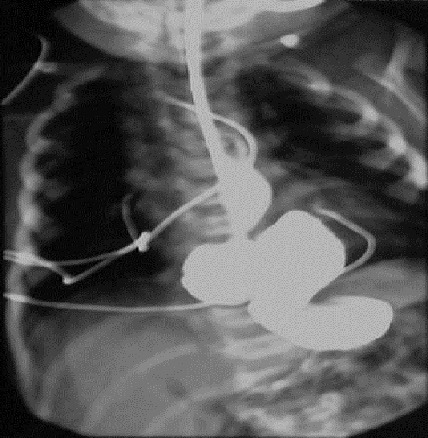
Upper gastrointestinal contrast study showing organoaxial volvulus of stomach

A 1-day old girl normally delivered after an uneventful pregnancy, weighing 3.200 Kg referred to us with a suspected diagnosis of esophageal atresia due to excessive salivation, regurgitation with an impossibility to pass a nasogastric tube. Physical examination did not reveal any overt signs, but an orogastric tube was arrested at 12 cm from the gum margin. Plain X-ray of the chest with orogastric tube in situ demonstrated the presence of tip of the tube at 6 thoracic vertebral level as well as the presence of an abnormal gas shadow in the chest (Figure 1). Subsequent contrast study ruled out esophageal atresia and demonstrated partial herniation of the stomach through a large hiatal hernia, and organoaxial gastic volvulus (Figure 2). The patient underwent emergency surgical repair via laparotomy because of distress respiratory and the risk of gastric perforation. At exploration, the stomach was found herniated into the chest through a large hiatal defect, twisted but not ischemic, gastroperitoneal attachments and gastric ligaments were absent with mobile spleen, there was no abdominal malformation. After untwisting, the stomach was reduced into the abdominal, the hiatal defect was repaired and a Thal fundoplication was performed, and a gastropexy was added to the procedure. The postoperative course was uncomplicated. At 1-year follow-up she is asymptomatic and growing well.

## Discussion

The normal gastric attachments within the abdomen include four ligaments: gastrohepatic, gastrophrenic, gastrosplenic and gastrocolic, these attachments result in the relative fixation of the stomach in the left upper quadrant, and intact diaphragmatic crus prevent herniation. For intrathoracic herniation of stomach to occur, the ligamentous attachements must be lax and the diaphragmatic hiatus must be deficient [1], and congenital esophageal shortening is also a feature of some infants with intrathoracic stomach. This abnormality predispose to a sliding hiatus hernia as opposed to a herniation secondary rotation or volvulus of the stomach [2, 3]. Gastric volvulus is defined as an abnormal rotation of the stomach of more than 180, creating a closed loop obstruction that can result incarceration and strangulation. According to the axis around which the stomach rotates, gastric volvulus is classified as follows [4]: organoaxial, the stomach rotates around an axis that connects the gastroesophageal junction and pylorus. The antrum rotates in opposite direction to the fundus of the stomach, this is the most common type in both children and is usually associated with diaphragmatic defects, strangulation and necrosis commonly occur with this type. Mesentericoaxial is the type in which the axis bisects both lesser and greater curvatures. The antrum rotates anteriorly and superiorly so that the posterior surface of the stomach lies anteriorly, the rotation is usually incomplete and occurs intermittently. Vascular compromise is uncommon. Patients with this type usually present without diaphragmatic defects and usually chronic symptoms. The third type of volvulus is a combination of the first and second type in which the stomach twists both mesentericoaxially and organoaxially. In the majority of neonates with gastric volvulus the stomach remains in the abdominal cavity, although a gastric volvulus also can occur into the chest. According to etiology, gastric volvulus can be classified as either type 1(idiopathic) or type 2 (congenital or acquired) [4]. Some of the anomalies associated with gastric volvulus included congenital diaphragmatic hernia, wandering spleen, diaphragmatic eventration and asplenia-polysplenia syndrome, the gastroperitoneal attachments and gastric ligaments are often absent in patient with there anomalies making them susceptible to gastric volvulus. A variety of symptom may occur, but it is common that the symptom complex is indicative of upper alimentary tract obstruction, with or without cyanotic episode. In patient with intrathoracic gastric volvulus, the symptomatolgy may indicate an esophageal atresia as was suspected in our patient. In these neonates an orogastric tube gets arrested from the gum margin, a level lower than that usually seen in esophageal atresia [5]. Sever epigastric pain and distension, violent unproductive retching and inability to pass a nasogastric tube comprises the classical tried of borchardt [6], the features result from obstruction at the cardia and/or pylorus. Sawaguchi has attributed vomiting in young infants to the maldeveloppement of hiatal function. Intermittent or chronic gastric volvulus my cause diverse gastrointestinal and chest symptoms in children [7]

A plain X-ray of chest needs to be viewed carefully as the gastric shadow can be seen in lower chest, by the side of esophagus and separate from the pulmonary shadow. The contrast study will be performed for delineation of anatomy of esophagus and stomach, and a correct preoperative diagnosis could be made.

Acute gastric volvulus is a surgical emergency as delay in recognition and treatment can cause strangulation and perforation. Chronic volvulus however should be initially treated conservatively by keeping the patient in the prone position. Gastroscopic decompression for chronic gastric volvulus is also reported. Anterior gastropexy supplemented with a gastrostomy is a satisfactory solution to this life threatening problem. Percutaneous gastrostomy using anchoring devices and laparoscopic guided gastroscopy are newer modalities reported [8]. The need for an antireflux operation after hiatal hernia repair in patients with intrathoracic gastric volvulus remains controversial. [4]

## Conclusion

Intrathoracic gastric volvulus is seen at all ages through life, but rarely present in the neonatal period. The purpose of this paper is to draw attention to the possibility of gastric volvulus at the newborn and to suspect this disease entity when its inability to pass a nasogastric tube confused with esophageal atresia
